# Impacts of land cover change on the plant resources of an endangered pollinator

**DOI:** 10.7717/peerj.11990

**Published:** 2021-10-05

**Authors:** Emma P. Gómez-Ruiz, Thomas E. Lacher Jr, Antonio Moreno-Talamantes, José Juan Flores Maldonado

**Affiliations:** 1 Especies, Sociedad y Hábitat, A.C., Apodaca, Nuevo Leon, Mexico; 2 Department of Ecology and Conservation Biology, Texas A&M University, College Station, TX, United States of America

**Keywords:** *Leptonycteris nivalis*, Agave, Bats, Fragmentation, Land cover change, Mexico

## Abstract

One of the key drivers of pollinator declines is land cover change. We documented for the first time the impacts of over three decades of land cover change in Mexico on the plant resources of an endangered migratory pollinator, the Mexican long-nosed bat, *Leptonycteris nivalis*. This species is considered endangered under national and international criteria due to population declines over 50% in the past 10 years. Pregnant females of this bat species migrate every year following the blooms of *Agave* spp. from central Mexico to the southern United States; moving pollen over its 1,200 km long migratory corridor and pollinating distant populations of *Agave* spp. Increases in human populations density and agricultural expansion may be reducing agave habitat over time. The objective of our study is to understand the land cover change trends in the northern range of the bat and identify potential fragmentation patterns in the region. We analyzed changes that occurred in three vegetation types where agaves are found in five time periods 1985, 1993, 2002, 2007 and 2011. The area of the three vegetation types selected was reduced by using only the overlap with potential agave habitat created with ecological niche modeling algorithms to obtain the available agave habitat. We then calculated fragmentation metrics for each period. We found a significant portion of habitat lost mainly due to expansion in agriculture. The total number of patches increased after 1985. Only 9% of the available agave habitat in 2011 is inside the limits of protected areas. We recommend restoring agave populations in depleted areas to help prevent soil erosion and provide multiple socio-economic benefits for the region in the short term, and, in the long-term maintaining foraging resources for nectar-feeding bats.

## Introduction

Human impacts on planetary biodiversity have increased greatly over the past century, prompting some conservationists to propose that we have entered a defining new period, the Anthropocene (Lewis & Maslin, 2015). Declines in biodiversity vary across taxa and are driven by multiple threats, including habitat loss and fragmentation, climate change, and overconsumption (Lacher & Roach, 2018). All these factors combine to varying degrees, and impact species at different spatial and temporal scales, altering important ecosystem functions (Lacher Jr et al., 2019). To maintain and restore important ecosystem processes, we need to understand the threats at the local and regional scale in order to implement effective conservation actions (Rands et al., 2010). There is agreement that habitat loss because of human-induced land cover change is currently the most important factor contributing to biodiversity declines in Earth’s terrestrial ecosystems (Millenium Ecosystem Assessment, 2005; Newbold et al., 2015; Chaudhary & Mooers, 2018).

A rising concern is the decline in pollinators because of land cover change (Tscharntke et al., 2005; Winfree, Gross & Kremen, 2011; Senapathi et al., 2015). Animal-mediated pollination contributes to the production of goods for humans (Lacher Jr et al., 2019). It also reinforces the reproduction of wild plants on which other services or service-providing organisms depend (Kremen et al., 2007). The reproductive fitness of a plant depends substantially on the number of pollen grains transferred (Hildesheim et al., 2019). An effective pollinator can transport the most pollen grains possible. Flowering plants have evolved flower characteristics that attract the most effective pollinators (Fenster et al., 2004). Flowers of plants of the genus *Agave*, subgenus *Agave* (hereafter agaves), are large and showy, white, or light colored, have strong odors and produce more nectar during the night (Gentry, 1982). These characteristics attract nectar-feeding bats, and for some agave species bats are more efficient pollinators than birds and insects (Arizaga et al., 2000; Molina-Freaner & Eguiarte, 2003; Arias-Cóyotl, Stoner & Casas, 2006). Bats are large-bodied, compared with other pollinators, and can carry greater pollen loads across distant populations of agaves (Fleming, Geiselman & Kress, 2009).

Agaves are important plants in arid and semiarid ecosystems because they help to prevent soil degradation (González-Elizondo et al., 2009). In these ecosystems, food resources for nectar-feeding animals are scarce, and agave flowers are a key food source for several species of insects, birds, and mammals (Rocha et al., 2006). Moreover, agaves have economic and cultural value for humans, as they have been used for centuries in several cultures for food, fiber, and the production of commercial products like mezcal and tequila (Gentry, 1982; Colunga-García et al., 2007).

Among the nectar-feeding bats that pollinate agaves, the Mexican long-nosed bat (*Leptonycteris nivalis*) is capable of moving pollen over long distances along its 1,200 km long migratory corridor (Fleming, Geiselman & Kress, 2009). *L. nivalis* migrates every spring from central Mexico to the southern United States following the blooms of agaves (Moreno-Valdez, Grant & Honeycutt, 2000; Gómez-Ruiz & Lacher, 2017). In the northern portion of this bat’s range, particularly the states of Coahuila, Nuevo León, Zacatecas, Tamaulipas, and San Luis Potosí, agaves are visited by several other species of nectar-feeding bats and moths, but agaves are the primary food source of *L. nivalis* during the migration (Arita & Santos-del Prado, 1999; Moreno-Valdez, Honeycutt & Grant, 2004; England, 2012; Burke et al., 2019).

*L. nivalis* is considered endangered under national (US and Mexico) and international (International Union for the Conservation of Nature) criteria (US Fish and Wildlife Service, 1988; SEMARNAT, 2010; Medellín, 2016). Its populations have declined by more than 50% over the past ten years (Medellín, 2016). Pregnant and lactating females are found in bat colonies in the northern range (Hensley & Wilkins, 1988; Moreno-Valdez, Honeycutt & Grant, 2004; Adams, 2016). Researchers have suggested that pregnant females give birth in northern Mexico prior to their arrival in Emory cave at Big Bend National Park in Texas (Easterla, 1972). Therefore, it is a conservation priority to maintain foraging resources in the northern range of *L. nivalis*.

Among terrestrial habitats, arid and semi-arid lands are under particular threat due to degradation and fragmentation associated with grazing and agriculture (Galvin et al., 2008). This has also been a recent trend in northern Mexico, much of this driven by urbanization and agriculture (Pérez Miranda et al., 2012). The objective of this analysis is to assess the degree of land-cover change in the northern range of *L. nivalis*, and to quantify trends in the distribution of dry forest and desert scrub habitats where agaves occur. We hypothesize that increasing human population density and associated agricultural and livestock expansion have reduced available agave habitat, impacting both the availability of resources for *L. nivalis* and traditional human livelihoods in the region. In this study we used geographic information system tools to analyze the effects of three decades of land cover change (LUCC) on the potential area of distribution of *Agave* species occurring in the northern Mexican portion of the bat’s range. We identified fragmentation trends and suggest conservation opportunities to help maintain the endangered *L. nivalis*–*Agave* pollination corridor.

## Materials & Methods

**Figure 1:**
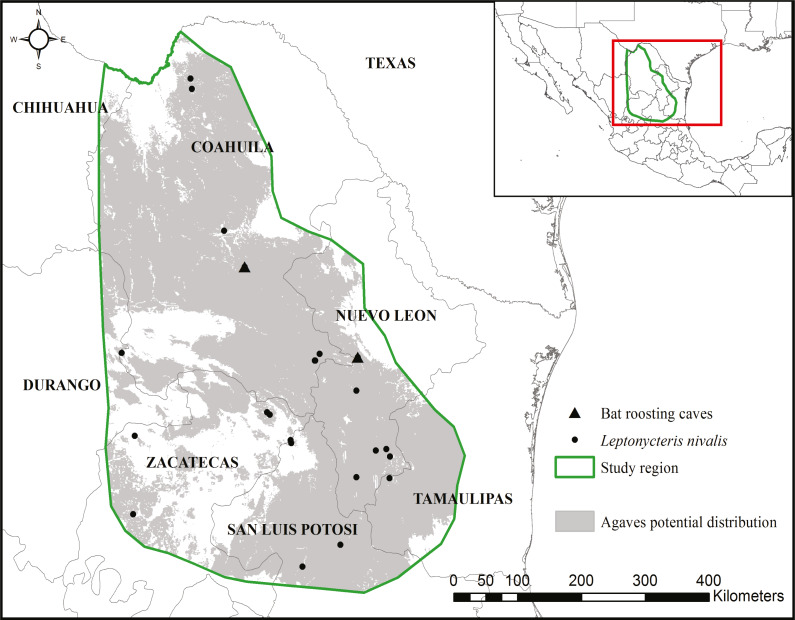
Study region. The region was delimited considering the potential distribution of nine *Agave* spp. (created in Gómez-Ruiz & Lacher, 2017) and known occurrences of the bat *Leptonycteris nivalis*. The region extends from top left 29.129238°, −103.562277° to bottom right 22.247175°, −99.285731°. Land cover maps are publicly available at INEGI’s website: www.inegi.org.mx.

The study area encompassed the potential distribution of the *Agave* species that form the *L. nivalis-Agave* pollination corridor (created in Gómez-Ruiz & Lacher (2017)). The northern portion of the corridor is where *L. nivalis* females give birth and where agaves are the main food source for the species (US Fish and Wildlife Service, 2018). We focused this analysis on the northern portion of the corridor within Mexico (Fig. 1). We focused on paniculate Agaves (subgenus Agave). There were 11 possible species in the region used in the analysis of the pollination corridor (Gómez-Ruiz & Lacher, 2017) which covered a much larger area, and nine were included in the analyses. The region selected for the analysis in this paper has at least eight confirmed species. The entire region used in this study is contained within the known points of occurrence and distribution of *L. nivalis* (Gómez-Ruiz & Lacher, 2017).

We used Land Use/Land Cover (LC) maps, scale 1:250 000, created by Mexico’s National Institute of Geography and Statistics (INEGI). To date, INEGI has produced LC maps at 1:250,000 scale for the following time periods: Series I 1985, Series II 1993, Series III 2002, Series IV 2007, and Series V 2011. These maps are available for download from INEGI’s website (http://www.inegi.org.mx) in vector format and are the best available information on land use and land cover for Mexico at a regional scale. We used the software ArcGIS 10.2^®^ to convert the LC maps to raster format with a pixel size of 100 m and projected them to North America Albers Equal Area Conic to estimate areas correctly. The Land Use/Land Cover classes used by INEGI in each period varies due to adjustments in the methodologies and the use of new types of interpretable inputs (aerial photography, satellite imagery, etc.) therefore we reclassified into only nine classes of interest (Table 1).

**Table 1:** Reclassification of land use and land cover maps, scale 1: 250 000 (INEGI).

Generalized land cover class	CODE	Description
Agriculture	AA	All types: seasonal, irrigation, cultivated grasslands, silviculture
Conifer forest	BC	Types: pine, pine-oak, and forest with *Pseudotsuga* sp. or *Picea* spp.
Deciduous forest	BD	Types: oak, oak-pine, and riparian forest with one or more of the following tree species: *Taxodium mucronatum*, *Salix* spp., *Fraxinus* spp., *Populus* spp., *Platanus* spp. and *Astianthus viminalis*
Water	H2O	Natural and man-managed water reservoirs
Other	NI	Vegetation types that are not *Agave* habitat
Bare ground	SV	Areas without vegetation naturally or due to anthropogenic influence
Desert scrub	VM	Succulent shrub, shrubs dominated by rosette plants (*Agave* spp, *Yucca* spp., *Dasylirion* spp.), creosote bush (*Larrea* spp.), shrub dominated by *Helietta parvifolia* and/or *Acacia berlandieri*.
Grassland	VP	All types of natural grasslands
Human settlements	ZU	Cities, Towns, Infrastructure

We used the Land Change Modeler for ArcGIS extension (2.0) (Clark Labs^®^) and the software ENVI 5.2^®^ to obtain land cover change statistics and transition matrices. In addition, we produced change maps of the following vegetation classes where agaves occur: desert scrub, conifer and deciduous forest. We calculated percent change for each class. Percent change is the area for a class divided by the area of a class in the later cover image and multiplied by 100.

To identify potential fragmentation trends on the agave corridor, we created maps of available agave habitat for each time period, identifying the overlap of the potential agave habitat created in previous studies (Gómez-Ruiz & Lacher, 2017), with the areas of the generalized land cover classes where wild agaves occur. Other studies have used ecological niche models to evaluate the impacts of land cover change on species (Sánchez-Cordero et al., 2005; Peterson et al., 2006; Ríos-Muñoz & Navarro-Sigüenza, 2009; López-Arévalo et al., 2011; Yañez Arenas et al., 2012). First, we extracted from the LC maps the areas of the three generalized land cover classes where wild agaves occur (desert scrub, deciduous forest, conifer forest), and created a binary raster where the value of 1 indicated presence of the targeted classes. Next, we used the potential distribution maps of agaves and combined them into one binary raster where 1 indicated presence of at least one agave species, and 0 indicated absence of all species. Then we summed the binary raster obtained from the LC map and the binary raster obtained from the agave distribution models. We reclassified the output raster to create a binary raster where 1 indicated available agave habitat. This raster had a pixel resolution of 1 km. Finally, using the raster of available agave habitat, we calculated landscape metrics commonly used as indicators of fragmentation: total number of patches, mean patch size, and mean nearest neighbor distance. These metrics were obtained using Patch Analyst extension for ArcGIS (Rempel, Kaukinen & Carr, 2012).

Additionally, we estimated the amount of remaining agave habitat in the 2011 land cover map that is located within protected areas designated by Mexico’s national commission on protected areas (CONANP). In this study, we focused only on human- driven land-use changes and did not include other factors, such as climate change, that could affect the distribution of agaves.

## Results

**Figure 2:**
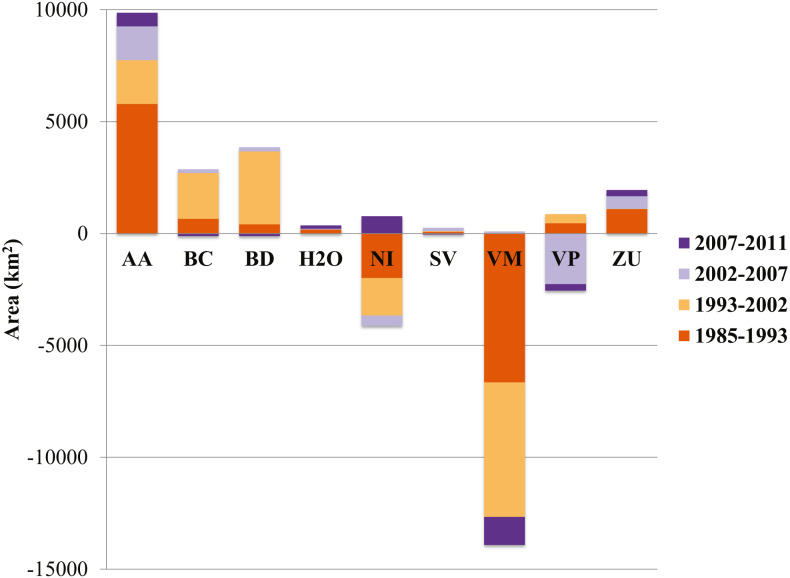
Area (km^2^) net change (original area + gain –loss) per land cover class. AA, Agriculture; BC, Conifer forest; BD, Deciduous forest; H2O, Water; NI, Other; SV, Bare ground; VM, Desert scrub; VP, Grassland; ZU, Human settlements.

**Figure 3:**
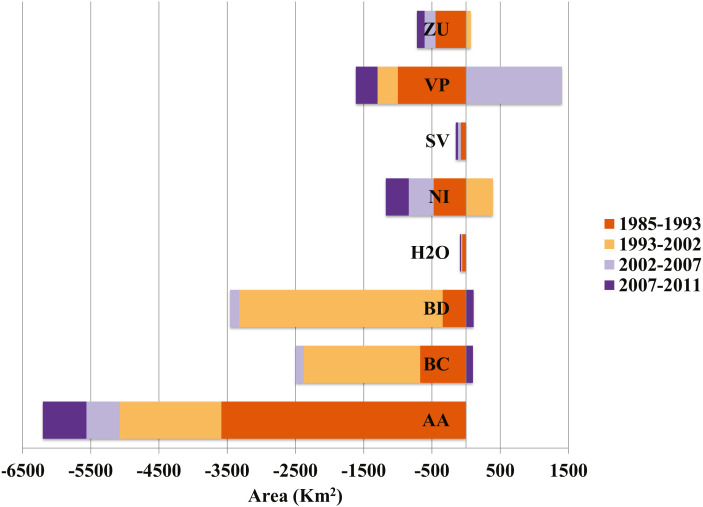
Contributions to net change in desert scrub. The horizontal axis indicates the area (km^2^) either gained (positive) or lost (negative) and to which class the area transformed. AA, Agriculture; BC, Conifer forest; BD, Deciduous forest; H2O, Water; NI, Other; SV, Bare ground; VM, Desert scrub; VP, Grassland; ZU, Human settlements.

Of the three generalized land cover classes where the agaves are found, desert scrub had the largest cover area in all five LC maps used (Table 2). The land cover change analysis indicates that desert scrub was the class with the largest negative net change from 1985 to 2011, and most of this change occurred between 1985 and 2002 (Fig. 2). The annual net loss in desert scrub area has decreased from the early time intervals (1985–1993) to the latest (2007–2011) (Table 3). The analysis of contributions to the net change in desert scrub shows that most of the area transitioned to agriculture (Fig. 3).

**Table 2:** Total area (km^2^) for each land cover class in the different LUCC maps.

Generalized land cover class	Series I 1985	Series II 1993	Series III 2002	Series IV 2007	Series V 2011	Total difference between 1985 and 2011
AA	32,188	37,979	39,932	41,449	42,054	9,866
**BC**	**7,614**	**8,276**	**10,320**	**10,487**	**10,367**	**2,753**
**BD**	**7,983**	**8,388**	**11,650**	**11,835**	**11,720**	**3,737**
H2O	289	464	449	485	643	354
NI	28,323	26,371	24,688	24,230	25,007	−3,316
SV	648.58	714.48	750.84	904.83	861.81	213
**VM**	**182,648**	**176,002**	**169,982**	**170,073**	**168,826**	**−13,822**
VP	22,858	23,313	23,721	21,457	21,172	−1,686
ZU	214	1,299	1,310	1,882	2,155	1,941

**Notes.**

AA Agriculture BC Conifer forest BD Deciduous forest H2O Water NI Other SV Bare ground VM Desert scrub VP Grassland ZU Human settlements Agave habitats highlighted in bold

**Table 3:** Annual net change in area (km^2^). Positive numbers indicate a gain, negative numbers indicate a loss.

	Class			
Image intervals	AA	BC	BD	VM
1985–1993	724	83	51	−832
1993–2002	217	227	363	−669
2002–2007	303	33	37	18
2007–2011	151	−30	−29	−312

**Notes.**

AA Agriculture BC Conifer Forest BD Deciduous Forest VM Desert scrub

The desert scrub area loss between 1985 and 1993 occurred in areas located in the central eastern portion of Coahuila adjacent to Nuevo Leon; and the area loss between 1993 and 2002 occurred in areas located in the northern portion Coahuila within the study area.

**Figure 4:**
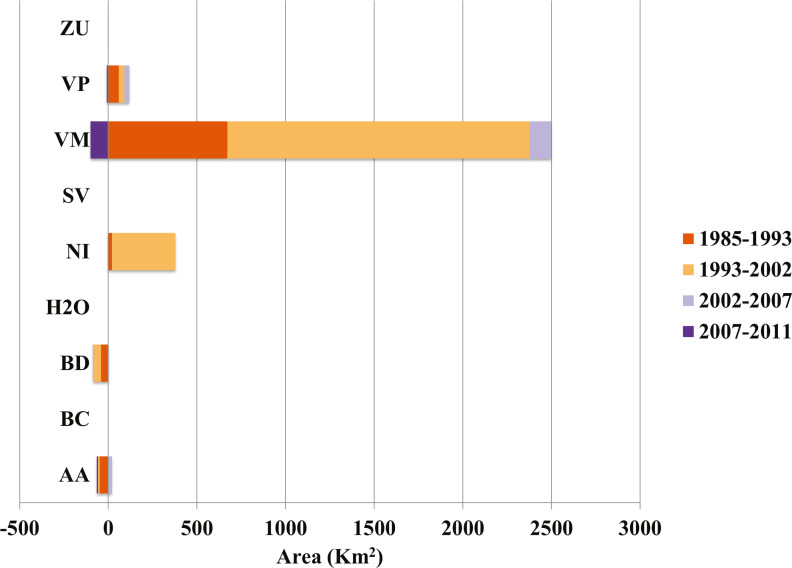
Contributions to net change in conifer forest. AA, Agriculture; BC, Conifer forest; BD, Deciduous forest; H2O, Water; NI, Other; SV, Bare ground; VM, Desert scrub; VP, Grassland; ZU, Human settlements.

**Figure 5:**
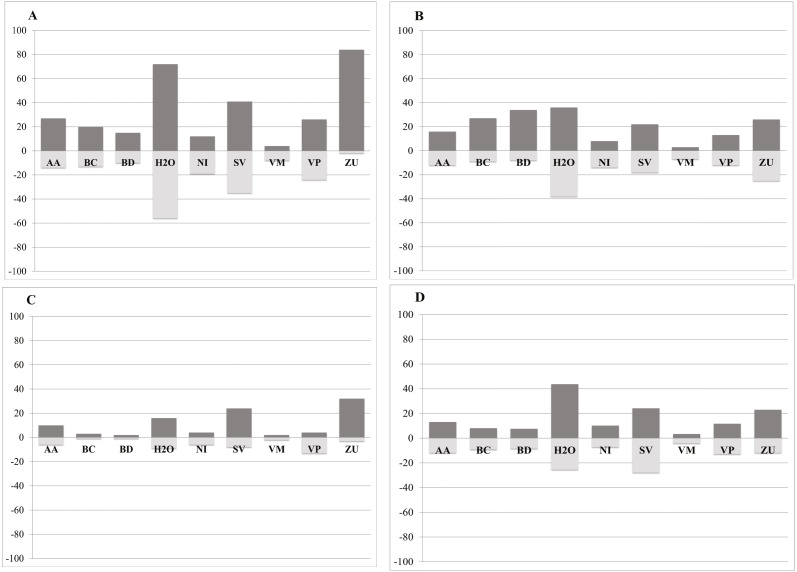
Percent change in gains (dark grey) and losses (light grey) per class for each time period: A = 1985–1993, B = 1993–2002, C = 2002–2007, D = 2007–2011. AA, Agriculture; BC, Conifer forest; BD, Deciduous forest; H2O, Water; NI, Other; SV, Bare ground; VM, Desert scrub; VP, Grassland; ZU, Human settlements *Percent Change = (# Pixels changed for a class/area of a class in the later land cover image)*100.

Our analysis indicates that conifer forest and deciduous forest increased mainly between 1993 and 2002 (Fig. 2, Table 3). Deciduous forest increased primarily in the northeastern portion of the state of Coahuila, within the study area; and the increase in conifer forest is largely observed in the sierras between the state of Coahuila and Nuevo Leon (Supplemental Information). Desert scrub contributed the most to the increment in the two types of forest (Fig. 4). The class with the highest value of percent change was human settlements with 84% area increase between 1985 and 1993 (Fig. 5A, for reference Figs. 5B, 5C, 5D).

The available agave habitat for each time period shows a reduction of 2% from 1985 to 2011, and 9% of the remaining agave habitat in 2011 is within the boundaries of protected areas designated by Mexico’s commission on protected areas (CONANP). The landscape metrics calculated indicate an increase in the number of habitat patches after 1985. The largest mean patch size was observed in 1985 to 1993, though the number remains fairly stable after 1993. The mean nearest neighbor distance between patches remained similar in all the time periods analyzed (Table 4).

## Discussion

The vegetation of northeastern Mexico ranges from low elevation semi arid scrib (Tamaulipan Biotic Province) and arid high elevation Chihuahuan desert (Lacher, 1999) to a complex of woody formations including mid-elevation Mexican shrublands and high elevation oak and pine forests (Valiente-Banuet & Verdú, 2020). There is exceptional diversity in the region due to the complex interaction of these vegetation formations, and regions like the Mexican shrublands are among the most poorly studied of Mexico’s ecosystems (Valiente-Banuet & Verdú, 2020). These regions also show high levels of fragmentation in a recent mapping exercise (Haddad et al., 2015) resulting in a high likelihood of future local species loss through extinction debt (Halley et al., 2016). This fragmentation can have severe negative consequences on ecosystem function through the loss of habitat specialists and the increase in generalists once critical thresholds in available habitat are reached (Pardini, Nichols & Püttker, 2018).

**Table 4:** Landscape metrics for each land cover map. Standard error shown in parenthesis.

Land cover map	Number of patches	Mean patch size (km^2^)	Proportion of landscape area	Mean nearest neighbor distance (km)
I (1985)	711	21.52 (19.63)	77.22	1.55
II (1993)	783	19.04 (17.08)	77.42	1.54
III (2002)	780	19.11 (17.08)	77.70	1.54
IV (2007)	760	19.73 (17.82)	77.99	1.53
V (2011)	740	20.15 (18.17)	78.14	1.54

Pollination is an important ecosystem function known to be sensitive to habitat loss and high levels of fragmentation (Kolb, 2008; Hadley et al., 2014). We centered our analysis on the changes in the three classes where agaves occur in the study region: desert scrub, deciduous forest, and conifer forest. Within our study area, the LUCC analysis indicates that loss in desert scrub occurred primarily between 1985 and 1993 and occurred in the central eastern portion of Coahuila adjacent to Nuevo Leon, probably due to the expansion of the main urban areas in the region (Monterrey, Saltillo, and Monclova) along with agricultural activities around the cities. This may indicate that at the regional level the cause of the LUCC may vary, depending on the land use dynamics of each region.

Our findings on the degradation of desert scrub are consistent with other LUCC studies for the study region (Pérez Miranda et al., 2012; Trucios Caciano et al., 2012). Desert scrub is the most common vegetation type in Mexico, occupying 26.2% of the territory (CONABIO, 2009). By 2011, 10% (5.8 million hectares) of the desert scrub area was transformed, and the main cause of this transformation is the expansion of agricultural areas (SEMARNAT, 2013). These results were also observed, albeit in a much broader scale analysis, by Bonilla-Moheno & Aide (2020). They note agricultural expansion in the Chihuahuan ecoregion, precisely where we also observed these changes on a finer scale.

Our results also indicate an increase in deciduous and conifer forest replacing areas previously occupied by desert scrub, a trend also noted in another recent analysis (Bonilla-Moheno & Aide, 2020). This finding is expected considering the possibility of natural forest succession over the time lapse of our analysis. In the desert scrub category, we included scrublands, which are found adjacent to forest in the foothills of mountains, and it is possible that forest extended into those areas. Other land cover change studies in Mexico report similar trends, showing an increase in forest areas (Moreno-Talamantes & Garcia-Aranda, 2012; Trucios Caciano et al., 2012). The gain in deciduous forest between 1993 and 2002 in areas located in northern Coahuila could have been the result of the implementation of conservation activities in the area after the creation of the federal protected area Maderas del Carmen in 1994 (CONANP, 2008).

The increase in number of available agave habitat patches since 1985 suggests a fragmentation trend. There were three land-cover classes in the fragmentation analysis: conifer forest, deciduous forest, and desert scrub. The fragmentation statistics show a nearly constant nearest neighbor distance over time and a fairly stable number of landscape patches of habitats supporting agaves. This was likely the result of increases in conifer and deciduous forest land cover and patches from 1985 to 2002 with large declines in desert scrub from 1985 to 2002, so agave habitat patches were replacing each other. From 2002 to 2011 numbers were similar except for a large jump in the loss of desert scrub habitat in 2007-2011. As desert scrub is the most representative habitat for agaves in the study region, fragmentation statistics of agave habitat classes alone do not reflect the degree of loss of agaves in the region.

The increase in human settlements after 1985 in the region was likely the result of migration of people seeking employment opportunities with the growing industries (*e.g.*, mining, energy, manufacturing, construction) in the main cities of the region: Monterrey, Saltillo, and Monclova. Since the 1970s, Mexico has seen an urbanization trend, with approximately 77% of the total population currently living in cities. The abandonment of rural areas reduces the local pressure on the land use and has been related to natural vegetation recovery in several countries, including Mexico (Bonilla-Moheno, Aide & Clark, 2012). The abandonment of rural areas in the study region could have contributed to the increase in forest areas.

Human productive activities (agriculture, livestock, and urban development) are still the most important contributor to habitat loss and subsequent fragmentation. Agriculture and cattle ranching are the main driving forces of the degradation of ecosystems in arid regions, such as our study region (SEMARNAT, 2019). Still, there are other human activities that are threatening this region, such as mining (especially coal mining, shale gas extraction, and material banks). Mining has been an important economic activity since the early 1900s, and significant portions of the landscape have been directly and indirectly affected by this activity, by degradation of the vegetation and pollution of the soil (Marroquín-Castillo et al., 2017). Mining activities are indirectly included in the land cover maps used in this study, particularly the areas affected by open pit mining which are classified as bare ground and mining facilities as human settlement.

Moreover, significant portions of the region have been affected by severe drought and fire. Between 1998 and 2017, the state of Coahuila had the second largest proportion of area affected by fires in the country, with more than 598 000 hectares burned (SEMARNAT, 2019). Most of this area is located within our study area. The effect of fire on the agave species present in our study area has not been studied, however, agave traits such as succulent leaves and a thick cuticle decrease flammability and facilitate fire resistance (Rodríguez-Trejo, Pausas & Miranda-Moreno, 2019). Studies in other regions indicate low mortality of agave after fire. Slauson (2002) reports 3.3% mortality after fire for *Agave palmeri*. Rodríguez-Trejo, Pausas & Miranda-Moreno (2019) reports 10% mortality for *Agave potatorum*.

During our field surveys in 2012 and 2013, we observed the effects of the severe drought that occurred in 2011 affecting large areas in northern Mexico. The drought affected extensive cattle ranching activities because there was not enough forage available for the cattle and, as an alternative, the cows foraged on any vegetation available, including agaves, increasing the level of degradation.

The recovery of depleted vegetation in arid lands is slow. For instance, agaves are slow-growing plants that bloom only at the end of their life cycle at 15–20 or more years (Gentry, 1982). The migration of the endangered *L. nivalis* relies on the agave blooming events (Gómez-Ruiz & Lacher, 2017). To have blooming agave every year, there needs to be agaves in late life stages. Even if depleted areas are restored with agaves, there will be a time lag for blooming events to occur meaning that restoration needs to happen as soon as possible to account for this lag. The fragmentation and disturbance on the available agave habitat can result in the lack of mature agave to produce flowers, and this would disrupt *L. nivalis* migration. Climate change is another factor that would affect the distribution of agave habitat (Gómez-Ruiz & Lacher Jr, 2019), however for this study we focused on direct human-driven land-use changes.

An additional but unrelated threat to the migratory *L. nivalis* in the study region is the development of wind farms (Hernández-Escobedo et al., 2014). Currently, wind turbines are operating in areas dominated by desert scrub in Coahuila, Nuevo Leon and Tamaulipas, sites of high potential for wind energy development (Wood et al., 2012) within the potential distribution of the migratory corridor (Moreno-Talamantes & Tovar, 2018). Large numbers of bats are killed at wind energy facilities in North America (Hammerson et al., 2017; Frick, Kingston & Flanders, 2019), but this impact can possibly be mitigated (Arnett et al., 2016), particularly during the spring and summer months when the endangered *L. nivalis* migrates to the region (Gómez-Ruiz & Lacher, 2017; Burke et al., 2019).

## Conclusions

The consequences of the large-scale modification of terrestrial ecosystems are well documented and tropical and subtropical dry forests have been particularly heavily impacted (Miles et al., 2006; Watson et al., 2016). This makes the attainment of conservation goals and targets particularly challenging (Watson et al., 2016), all this complicated by the additional synergistic impacts of climate change and habitat loss (Mantyka-Pringle, Martin & Rhodes, 2012), in particular the documented impacts of climate-change on the *L. nivalis*-*Agave* complex in this region (Gómez-Ruiz & Lacher Jr, 2019). Overall, our results highlight the need for the implementation of conservation strategies to mitigate fragmentation and degradation in the *Agave*-*L. nivalis* corridor. The pressures on the land cover change are a result of actions performed by various actors (government, private sector, local communities). Conservation strategies should be developed with participation of all actors. Recent efforts to engage local actors for the protection of bats and their habitat in our study region have demonstrated the value of engaging regional NGOs and local communities (Gómez-Ruiz et al., 2015).

We recommend implementing restoration activities of agave populations in depleted areas. This would prevent soil erosion in the short term and would provide foraging resources for the endangered nectar–feeding bats in the long term and maintain the *Agave*-*L. nivalis* pollination corridor.

##  Supplemental Information

10.7717/peerj.11990/supp-1 Supplemental Information 1 Land cover maps and classes for the study area AA, Agriculture; BC, Conifer forest; BD, Deciduous forest; H2O, Water; NI, Other; SV, Bare ground; VM, Desert scrub; VP, Grassland; ZU, Human settlements.

10.7717/peerj.11990/supp-2 Supplemental Information 2 Distribution of area gain and loss in desert scrub for each of the land cover series used

10.7717/peerj.11990/supp-3 Supplemental Information 3 Distribution of area gain and loss in deciduous forest for each of the land cover series used

10.7717/peerj.11990/supp-4 Supplemental Information 4 Distribution of area gain and loss in conifer forest for each of the land cover series used

10.7717/peerj.11990/supp-5 Supplemental Information 5 Available agave habitat for each of the land cover series used in the study

## Additional Information and Declarations

### Data Availability

The following information was supplied regarding data availability: Agave models are available from Harvard Dataverse: Gómez-Ruiz, Emma, 2020, “Agave distribution models in Gomez-Ruiz & Lacher 2017”, https://doi.org/10.7910/DVN/1MMWXM, Harvard Dataverse, V1.
