# Supplementary figures and images for: Impacts of land cover change on the plant resources of an endangered pollinator
Source: PeerJ. 2021 Oct 5;9:e11990. doi: 10.7717/peerj.11990 (PMC8500086; doi:10.7717/peerj.11990)

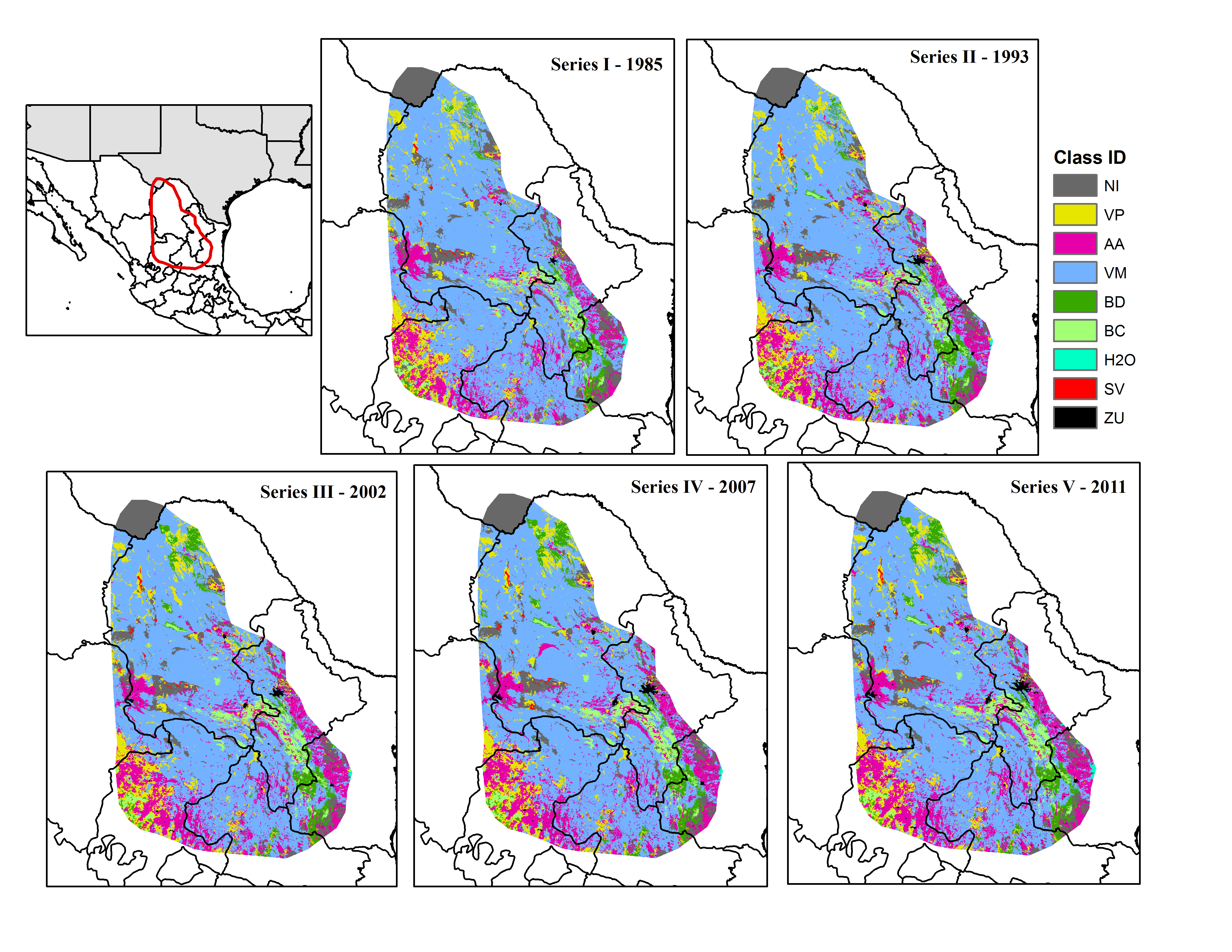

Supplement: Supplemental Information 1 — AA, Agriculture; BC, Conifer forest; BD, Deciduous forest; H2O, Water; NI, Other; SV, Bare ground; VM, Desert scrub; VP, Grassland; ZU, Human settlements. [file peerj-09-11990-s001.png]

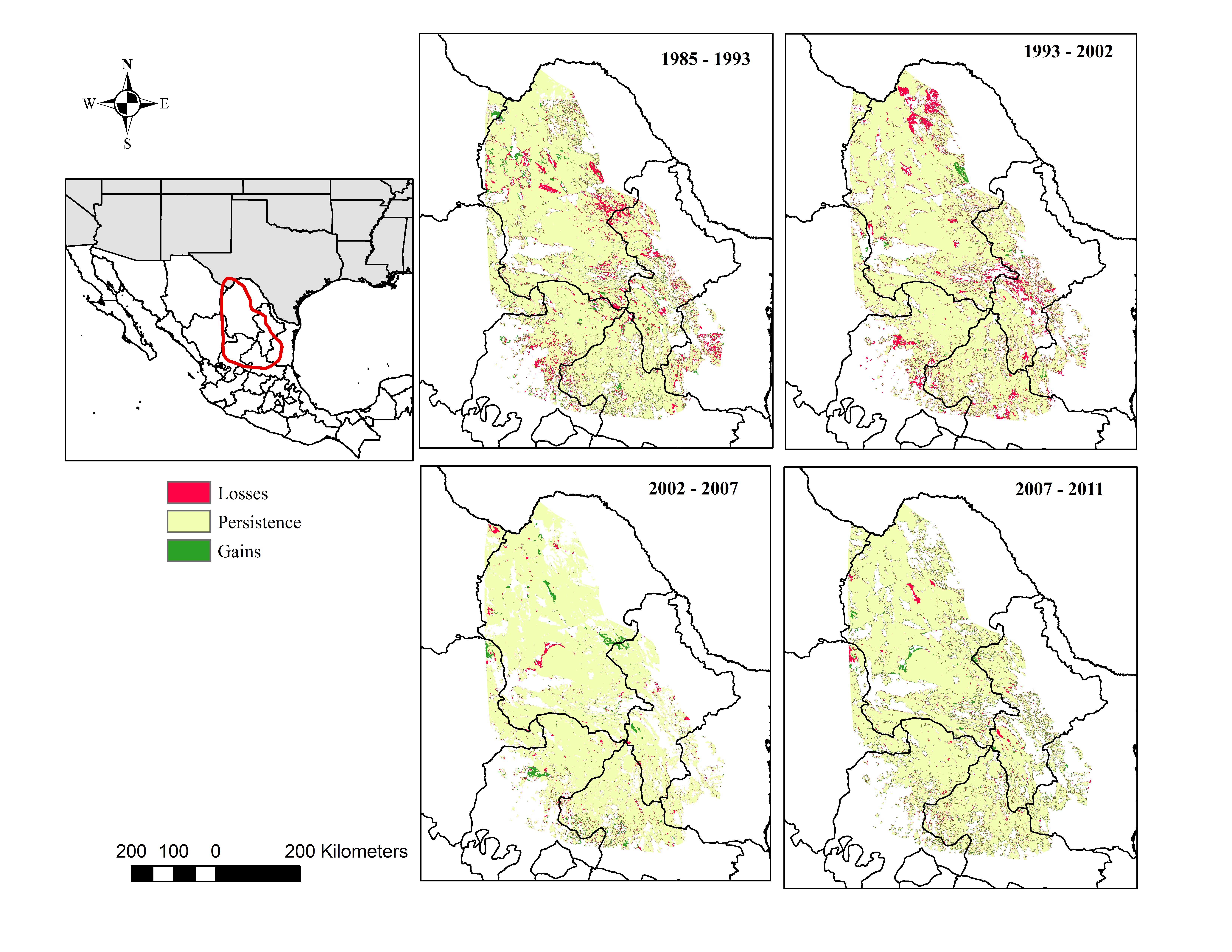

Supplement: Supplemental Information 2 [file peerj-09-11990-s002.png]

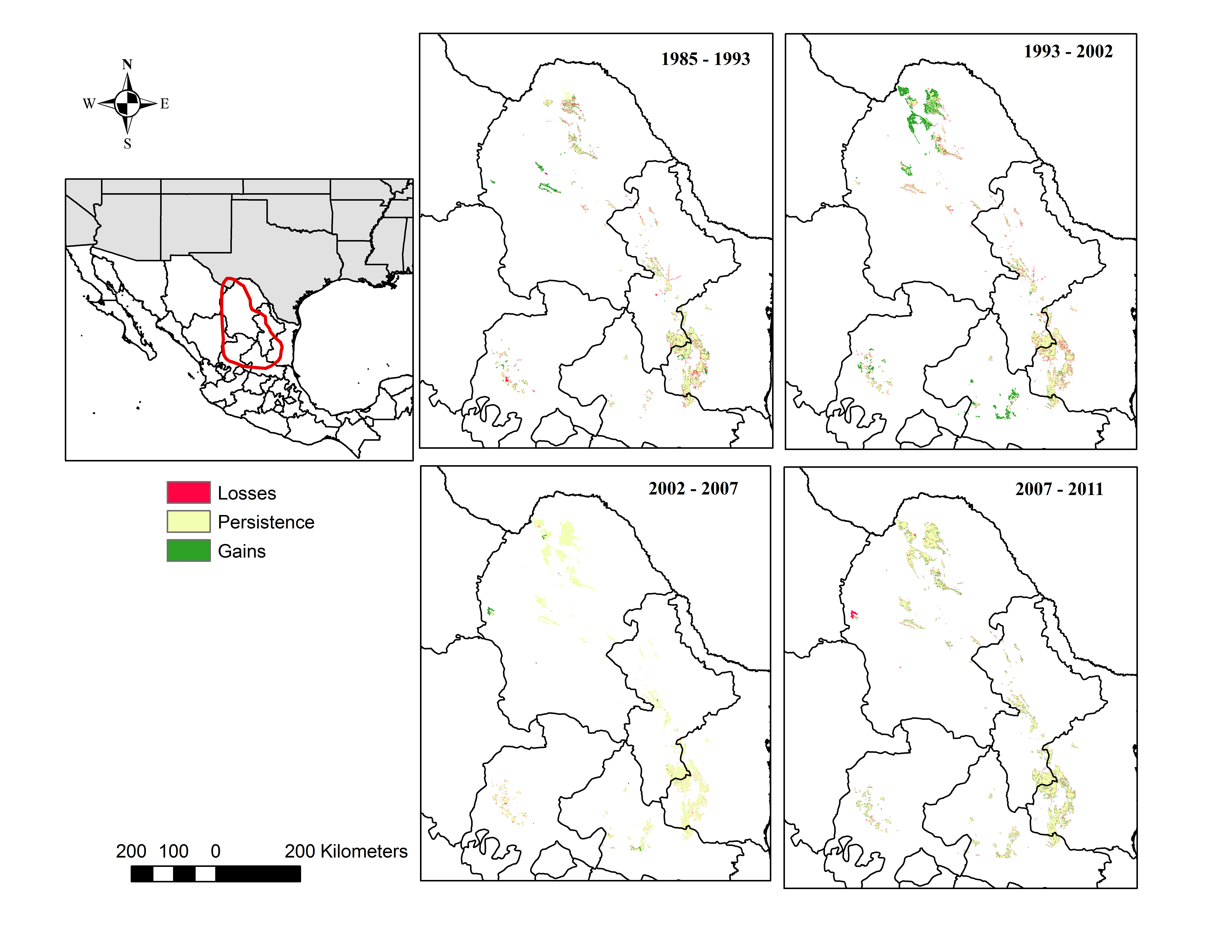

Supplement: Supplemental Information 3 [file peerj-09-11990-s003.png]

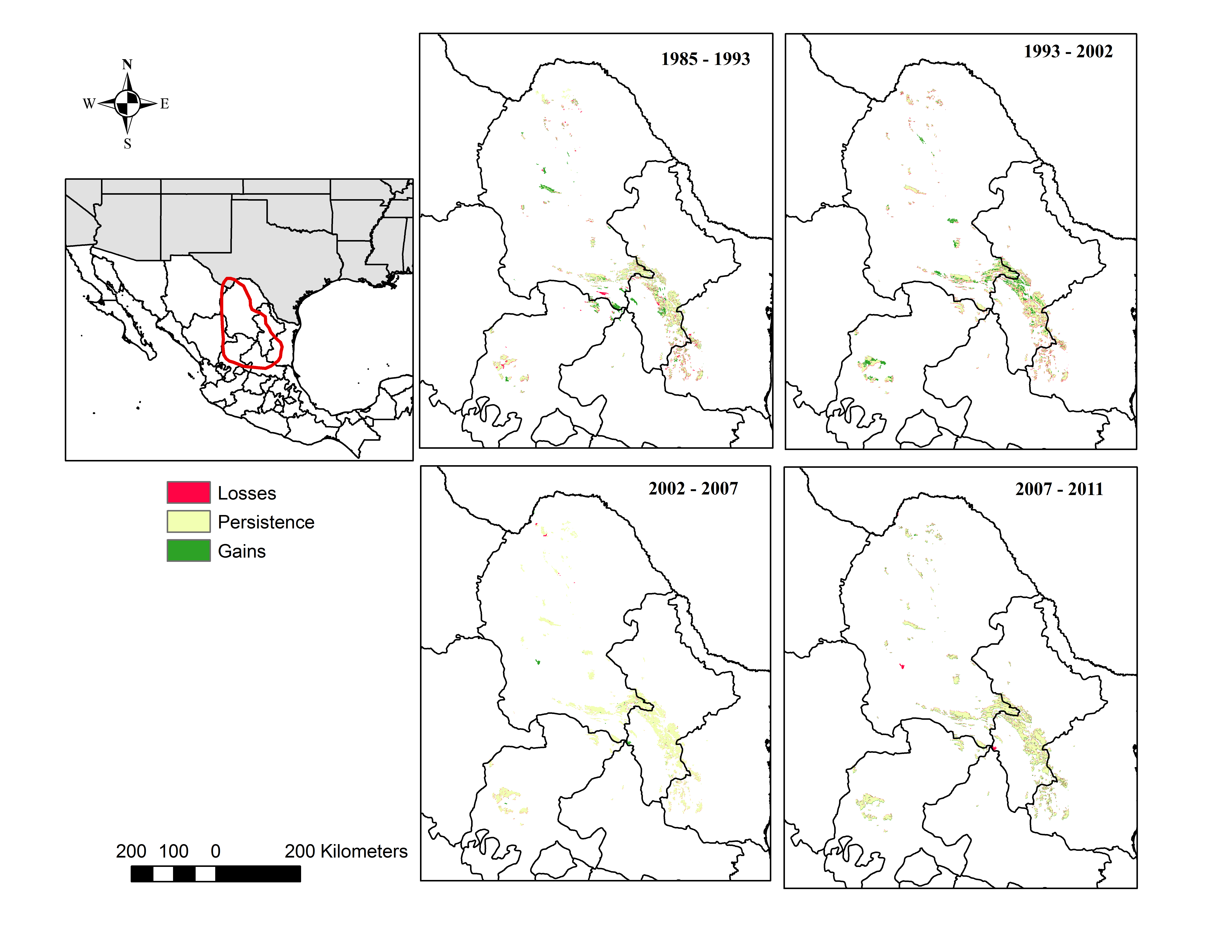

Supplement: Supplemental Information 4 [file peerj-09-11990-s004.png]

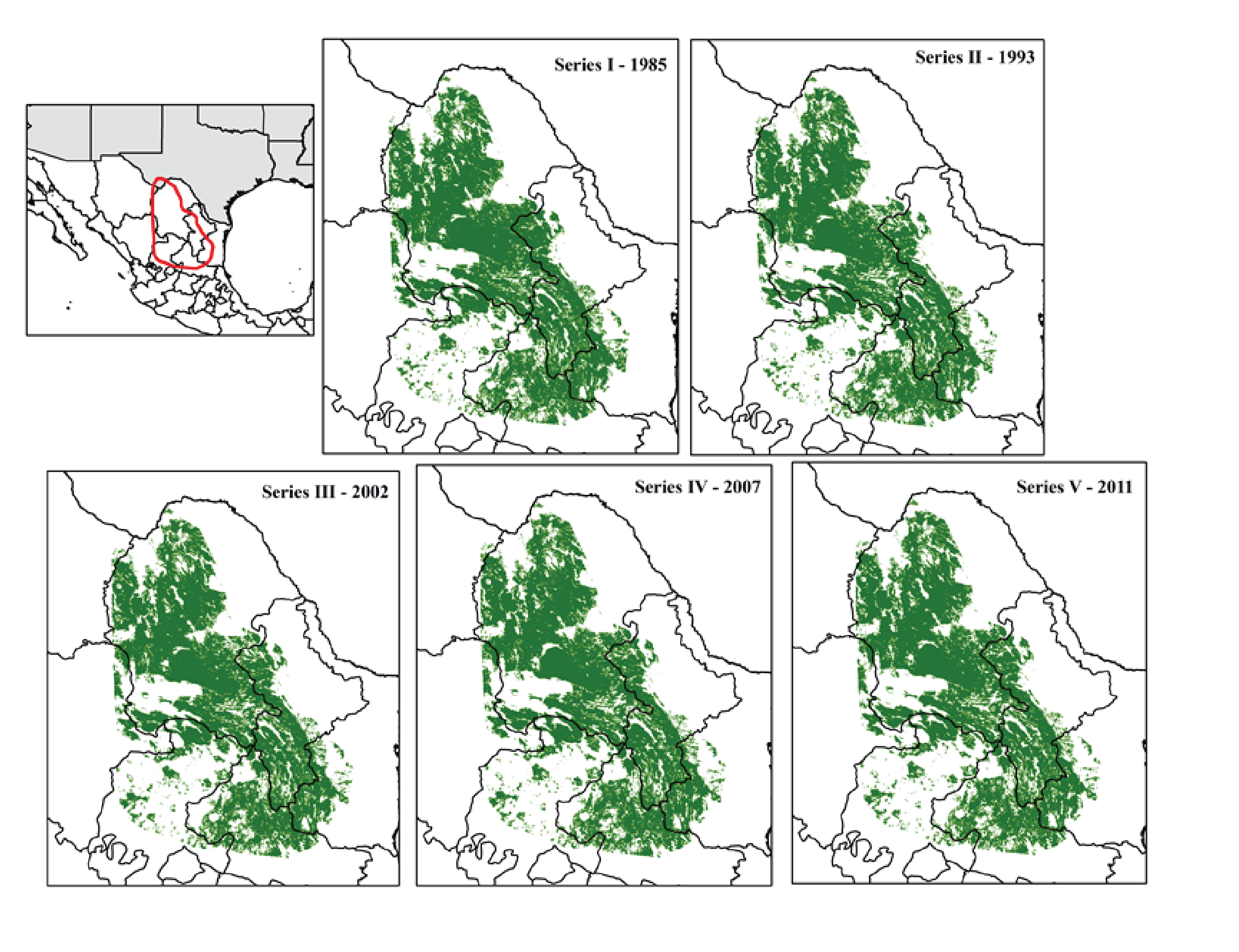

Supplement: Supplemental Information 5 [file peerj-09-11990-s005.png]
